# Detoxification technology and mechanism of processing with *Angelicae sinensis* radix in reducing the hepatotoxicity induced by rhizoma *Dioscoreae bulbiferae in vivo*

**DOI:** 10.3389/fphar.2022.984858

**Published:** 2022-09-28

**Authors:** Lingling Song, Junming Wang, Mingzhu Gong, Yueyue Zhang, Yamin Li, Xiaohui Wu, Lingyu Qin, Yaqian Duan

**Affiliations:** ^1^ College of Pharmacy, Henan University of Chinese Medicine, Zhengzhou, China; ^2^ Co-construction Collaborative Innovation Center for Chinese Medicine and Respiratory Diseases by Henan and Education Ministry of P. R. China, Henan University of Chinese Medicine, Zhengzhou, China

**Keywords:** optimized processing process, rhizoma dioscoreae bulbiferae, angelicae sinensis radix, hepatotoxicity, Nrf2 signaling pathway

## Abstract

Rhizoma Dioscoreae Bulbiferae (RDB) was effective on relieving cough and expectorant but accompanied by severe toxicity, especially in hepatotoxicity. A previous study found that processing with Angelicae Sinensis Radix (ASR) reduced RDB*-*induced hepatotoxicity. However, up to now, the optimized processing process of ASR-processed RDB has not been explored or optimized, and the detoxification mechanism is still unknown. This study evaluated the detoxification technology and possible mechanism of processing with ASR on RDB-induced hepatotoxicity. The optimized processing process of ASR-processed RDB was optimized by the content of diosbulbin B (DB), the levels of serum alanine aminotransferase (ALT), aspartate aminotransferase (AST), alkaline phosphatase (ALP), and histopathological analysis. The processing detoxification mechanism was evaluated by detecting the antioxidant levels of nuclear factor E2 related factor 2 (Nrf2) and its downstream heme oxygenase 1 (HO-1), quinone oxidoreductase 1 (NQO1), glutamylcysteine ligase catalytic subunit (GCLM), and the levels of downstream antioxidant factors of Nrf2. Besides, the antitussive and expectorant efficacy of RDB was also investigated. This work found that processing with ASR attenuated RDB-induced hepatotoxicity, which can be verified by reducing the levels of ALT, AST, and ALP, and reversing the pathological changes of liver histomorphology. And the optimized processing process of ASR-processed RDB is “processing at a mass ratio of 100:20 (RDB:ASR) and a temperature of 140°C for 10 min.” Further results corroborated that the intervention of processed products of ASR-processed RDB remarkably upregulated the Nrf2/HO-1/NQO1/GCLM protein expression levels in liver, and conserved antitussive and expectorant efficacy of RDB. The above findings comprehensively indicated that the optimized processing process of ASR-processed RDB was “processing at a mass ratio of 100:20 (RDB:ASR) and a temperature of 140°C for 10 min,” and the processing detoxification mechanism involved enhancing the level of Nrf2-mediated antioxidant defense in liver as a key target organ.

## Highlights

Processing with ASR alleviated serum transaminase levels caused by RDB.
Processing with ASR inhibitd the reduction of Nrf2 level caused by RDB.
Processing with ASR inhibitd liver lipid peroxidation caused by RDB.
Processing with ASR enhanced antioxidant levels caused by RDB.

## Introduction

Traditional Chinese medicine (TCM), the great treasure of the Chinese nation, plays an important value with its unique advantages (He et al., 2019; Luo et al., 2019; Jiang et al., 2020; Lin et al., 2020; Qiu et al., 2020; Wu et al., 2021). However, more recently, with the wide application of TCM, adverse reactions (like hepatotoxicity) have been attracted growing attention worldwide (Xie et al., 2020a; Liu et al., 2021; Zhang S. et al., 2021). Therefore, how to reduce the liver toxicity is the key link to ensure the safety of TCM (Chu et al., 2017; Zhou et al., 2018; Jin et al., 2019; Ruan et al., 2019; Deng et al., 2020; Zhang W. et al., 2021).

Rhizoma Dioscoreae Bulbiferae (RDB) was first recorded in the Song Dynasty “Kaibao Bencao” (Zhang et al., 2017), with a medicinal history of more than 1,000 years. Modern researches suggested that RDB has pharmacological effects such as anti-tumor (Wang et al., 2012; Cui et al., 2016), anti-inflammatory (Mbiantcha et al., 2011), antioxidant (Chaniad et al., 2020), regulating immune function (Cui et al., 2016), and is widely used in the treatment of hyperthyroidism (Jiang, 1978), colorectal carcinoma (Ahmad et al., 2018), and other diseases (Ghosh et al., 2012). However, it is often accompanied by strong liver toxicity in the process of medication, which has been confirmed in several clinical (Jiang, 1976; Jiang, 1981; Feng, 1989; Jin, 1996; Hua et al., 2011; Huang et al., 2013; Lu and Wu, 2014) and preclinical animal (Su et al., 2003; Ma et al., 2013; Sheng et al., 2014; Yang et al., 2016; Qu et al., 2019) studies. Since the hepatotoxicity induced by RDB severely limits the exertion and application of its outstanding curative effect, it is very urgent to carry out the detoxification research*.*

The “using Chinese botanical drugs to process other Chinese botanical drugs” method is the principle of processing the primary medicine by using a certain TCM as processing adjuvant in the processing technology, which could attenuate drug toxic reaction (Huang et al., 2011; Su et al., 2016; Shan et al., 2018; Jiang T. et al., 2021; Zhang K. et al., 2021; Zhang M. et al., 2021; Ota and Makino, 2022) or (and) increase the synergistic effect (Huang et al., 2011; Lu et al., 2018; Wang J. et al., 2019; Liao et al., 2021; Kong et al., 2022; Wang T. et al., 2022). Angelicae Sinensis Radix (ASR) is sweet in taste and warm in nature (Chen Q. et al., 2021), and has been proved to have a variety of pharmacological effects such as hepatoprotective (Hua et al., 2014), anti-oxidation (Nai et al., 2021), and its main pharmacological components have been proved to significantly reduce liver damage induced by carbon tetrachloride (Wang et al., 2020), ethanol (Wang et al., 1997), and concanavalin A (Wang et al., 2016). In addition, previous studies (Wu et al., 2020a; Wang C. et al., 2022) indicated that processing with ASR effectively reduced RDB*-*induced hepatotoxicity*.* However, so far, the optimized processing process of ASR-processed RDB has not been explored or optimized, and the detoxification mechanism is still unknown.

Given that, this study optimized the processing process of ASR-processed RDB through the detection of diosbulbin B (DB) content, serum biochemical indicators and liver histopathology, and explained its detoxification mechanism. Additionally, this study also discussed the antitussive and expectorant efficacy of RDB. This current research will contribute to provide support for the wide application of RDB, improve the safety and effectiveness of the medication.

## Materials and methods

### Plant materials

RDB (origin: Hubei) and ASR (origin: Gansu) were identified as the dried tubers of *Dioscorea bulbifera* L., the dried root of *Angelica sinensis* (Oliv.) Diels of the Apiaceae by Professor Chen Suiqing of School of Pharmacy, Henan University of Chinese medicine. The numbers of voucher specimens are in the order of RDB20180811, and ASR180601, respectively.

### Reagents and antibodies

Alanine aminotransferase (ALT), aspartate aminotransferase (AST), alkaline phosphatase (ALP), malondialdehyde (MDA), total superoxide dismutase (T-SOD), glutathione (GSH), glutathione peroxidase (GPx), glutathione transferase (GST) were all produced by Nanjing Jiancheng Bioengineering Institute (Nanjing, China). Antibodies such as nuclear factor-E2-related factor 2 (Nrf2), heme oxygenase-1 (HO-1), quinone oxidoreductase 1 (NQO1), glutamate cysteine ligase (GCLM) were obtained from GeneTex Inc. (San Antonio, United States). β-actin, and HRP-conjugated Affinipure Goat Anti-Rabbit lgG (H + L) were produced by Proteintech Group, Inc. (Chicago, United States). Antibody Lamin B was supported by Bioworld (Bioworld Technology Co. Ltd., Minnesota, United States).

Reference standard of DB was produced by Shanghai Yuanye Biotechnology Co., Ltd., (Shanghai, China). All chemicals and solvents were of chromatographic grade.

### Orthogonal experimental design of angelicae sinensis radix-processed rhizoma dioscoreae bulbiferae

The key factors in the processing process, such as the ratio of main drug (RDB) to adjuvant (ASR), processing temperature, and processing time, etc. were taken as the investigation factors. Under each factor, the three levels involved in the routine were taken as the inspection level, and the factor level table was designed according to the principle of orthogonal experimental design, as illustrated in Table 1.

**TABLE 1:** The L9 (3^4^) orthogonal experimental design table of ASR-processed RDB.

Factors/levels group	A. RDB: ASR (g)	B. Processing temperature (°C)	C. Processing time (min)
*A*_*1*_ *B*_*1*_ *C*_*1*_	100:5	120	10
*A*_*1*_ *B*_*2*_ *C*_*2*_	100:5	140	15
*A*_*1*_ *B*_*3*_ *C*_*3*_	100:5	160	20
*A*_*2*_ *B*_*1*_ *C*_*2*_	100:10	120	15
*A*_*2*_ *B*_*2*_ *C*_*3*_	100:10	140	20
*A*_*2*_ *B*_*3*_ *C*_*1*_	100:10	160	10
*A*_*3*_ *B*_*1*_ *C*_*3*_	100:20	120	20
*A*_*3*_ *B*_*2*_ *C*_*1*_	100:20	140	10
*A*_*3*_ *B*_*3*_ *C*_*2*_	100:20	160	15

### Preparation of rhizoma dioscoreae bulbiferae and processed products of angelicae sinensis radix-processed rhizoma dioscoreae bulbiferae

Different mass fractions of ASR were weighed and decocted for 30 min with water (1:10, w/v) to obtain the filtrate. Then, the dregs were boiled in water (1:8, w/v) for 20 min. Then, the filtrates were combined and concentrated to the corresponding volume (approximately 30 ml of ASR decoction was used for every 200 g of RDB).

An appropriate amount of RDB (e.g., 200 g) was taken and added the corresponding ASR decoction, respectively, according to the process combination in Table 1, mixed well, moisten, placed the drugs into the pan, processing them with different temperatures for the corresponding time, taken out, and dried to obtain the samples of processed products of RDB*.*

Processing RDB product (QC): The processing method was referred to the general processing rule 0213 method of Chinese Pharmacopoeia (Pharmacopoeia of the People's Republic of China, 2020). In brief, an appropriate amounts of RDB (e.g., 200 g) were weighed and placed in a preheated wok, processing them gently until they turns slightly brown (about 10 min), taken out and dried to get the processing RDB product.

Processing RDB with water (QSC): An appropriate amount of RDB (e.g., 200 g) was taken and added water (e.g., 30 ml), mixed well, and moisten (about 60 min), put the drugs into a preheated wok, processing gently until they were slightly brown (about 10 min), taken out and dried to get them.

ASR adjuvant group (ASR): An appropriate amount of ASR (such as 10 g) was taken, and added water (1:10, w/v) to decoct for 30 min, filter, the dregs were decocted in water (1:8, w/v) for 20 min, combined and centrifuged to obtain the supernatant, and concentrated to the corresponding volume.

### High-performance liquid chromatography analysis

Appropriate amounts of RDB and processed products of ASR-processed RDB (such as 10 g) were weighed and extracted with 95% ethanol for 2 times, 2 h/time, and filtered. Then, the filtrates were combined and concentrated under reduced pressure until there was no alcohol smell, and evaporated to dryness to obtain the extract, and the yield was 17.83%, 11.26%, 9.93%, 11.25%, 10.39%, 13.68%, 9.42%, 12.68%, 14.73%, 16.08%, 12.60%, and 10.65%, (the order was RDB*,* the nine processed products, QC, QSC) separately. A certain volume of extract (equivalent to 3 g of medicinal materials) was precisely weighed, added methanol to dissolve, and fixed volume to a 25 ml volumetric flask.

The HPLC method was as reported by the previous research, with slightly improved (Wu et al., 2020b). The system was Agilent 1,260 (Agilent Technologies, Inc., United States) and a C18 chromatographic column (250 mm × 4.60 mm, 5 μm, Agilent Technologies, Inc., United States) was selected. Acetonitrile-water (25:75) was used as the mobile phase. The flow rate was 1.0 ml/min^−1^, the column temperature was 30°C, and the detection wavelength was 210 nm.

### Animals

SPF-grade male ICR mice (weighing 18–22 g) were obtained from Shandong Experimental Animal Center (Shandong China), license NO. SYXK (Lu) 2020–0004. The mice were randomly given standard food and water, and kept under a specified environment, with a relative temperature of 21–23°C, the humidity of 60 %–65%, and a 12 h/12 h light/dark cycle, with lighting turned on at 7 a.m. every day. All the procedures were in strict accordance with the P.R. China’s legislation on the use and care of laboratory animals and approved by the Experimental Animal Ethical Committee of Henan University of Chinese Medicine (approval number: DWLL201903531).

### Experimental protocol

After 1 week of acclimating, 140 SPF-grade male mice were stochastically separated into the control group, RDB group, the above nine processed groups of ASR-processed RDB*,* QC, QSC, and ASR group (*n* = 10) based on their body weight. The RDB and the above-processed products were administered by gavage at the dose of 1.7 g/kg^−1^ for 14 days on the basis of the previous research (Wu et al., 2020b), and the gavage volume was 20 ml/kg^−1^. The ASR group was administered with a dose of 0.4 g/kg^−1^, and the control group was given an equal volume of 0.5% CMC-Na solution. After the last administration for 60 min, blood was taken from all mice, and after standing for 2 h, centrifuged to collect the upper serum. Then, the mice were sacrificed immediately, and the liver tissues of each group were quickly taken out and stored in a −80°C refrigerator.

### Determination of serum biochemical indexes in mice

The serum ALT, AST, and ALP levels were measured on the basis of the colorimetric method, Lai’s method, and sodium phenylene phosphate colorimetry method in the instructions of their respective kits, and calculated according to their corresponding formulas.

### Histopathological analysis

The left lobe of liver were fixed in 4% paraformaldehyde solution, embedded in conventional paraffin, and sliced at 4–5 μm. Sections were stained with hematoxylin-eosin (HE) for histopathological examination under microscope and photographed at 200 ×.

### Western blot

Liver proteins were detected by Western blot analysis. The extracted proteins were electrophoresis and transferred to PVDF membranes. After blocking with 5% skimmed milk powder, added the corresponding primary antibodies (rabbit anti-Nrf2: 1:500, rabbit anti-HO-1: 1:1,000, rabbit anti-NQO1: 1:1,000, rabbit anti-GCLM: 1:1,000) incubated overnight at 4°C. The next day, the membranes were washed with TBST for 4 times, each time/10 min. Then, HRP-conjugated affinipure goat anti-rabbit lgG (H + L) (1:5,000) was incubated for 1 h, washed again, and imaged with Tanon image software (Tianneng Technology Co., Ltd., Shanghai, China). The optical density (O.D.) of the immune contents was normalized to β-actin (total protein) or Lamin B (nuclear protein).

### Detection of lipid peroxidation and antioxidant levels in liver

The absorbance of liver tissue homogenate was detected by the thiobarbituric acid method in the instructions of the kit, and the MDA level was calculated by dividing the corresponding protein content of each group. The absorbance was measured based on the hydroxylamine method in the instructions of the kit, and then divided by the corresponding protein content of each group to calculate the T-SOD level in liver tissue. The levels of GSH, CPx, and GST were obtained by measuring the absorbance of liver tissue homogenate on the basis of the visible light colorimetric method in the kit instructions, and then dividing by the corresponding protein content of each group.

### Cough model induced by ammonia

80 SPF male ICR mice were separated into eight groups on the basis of their body weight (*n* = 10), the model group, RDB group, worst processing technology (*A*_*2*_ *B*_*2*_ *C*_*3*_) group, intermediate processing technology (*A*_*2*_ *B*_*3*_ *C*_*1*_) group, the optimized processing technology (*A*_*3*_ *B*_*2*_ *C*_*1*_) group, QC, QSC group, and positive drug Keke tablet group. The RDB and processed products of ASR-processed RDB were intragastrically administered with the same dose of 1.7 g/kg^−1^, and 1.4 g/kg^−1^ Keke tablets were given by gavage to the positive drug group based on the previous study (Wu et al., 2020b). All groups were given continuous intragastric administration for 14 days. The ammonia-induced cough model was carried out 60 min after the last administration. Briefly (Hu et al., 2021), the 500 ml beaker was placed on the desktop with 1/4 of the 10 cm × 10 cm qualitative filter paper, and 0.1 ml of concentrated ammonia water was injected into the filter paper with 1 ml syringe. After 30 s of natural volatilization, the filter paper was quickly extracted and put into the mice to be tested. The cough was judged by abdominal contraction, mouth opening, and cough sound of mice, and the cough incubation period of cough was the time from the beginning to the first cough in mice. The cough latency and the number of coughs in each group within 2 min were recorded, and the cough relieving rate was calculated. Cough relieving rate (%) = (the number of coughs in the concentrated ammonia water-induced cough group - the number of coughs in the administration group)/the number of coughs in the concentrated ammonia water-induced cough group × 100%.

### Effects on phenol red excretion from trachea of mice

The experimental grouping and administration method were referred to the cough experiment induced by concentrated ammonia water. Referred to the previous method, with slight modifications (Jiang et al., 2014). After the last administration for 30 min, 0.1 ml/10 g of 5% phenol red saline solution was intraperitoneally injected. The trachea with the same length from the thyroid cartilage to the tracheal branch was taken and placed in a centrifuge tube containing 2 ml of saline (NaCl 0.9%), and added 0.1 ml of 0.5% sodium bicarbonate solution. Then, the centrifuge tubes with trachea were ultrasonically treated, soaked, and centrifuged to obtain supernatant. The absorbance value was detected at 546 nm and substituted into the phenol red standard curve to calculate the concentration of phenol red, that is, the phenol red excretion of mouse trachea. Taking the excretion of phenol red in the model group as 100%, the percentage of red excretion percentage of the other groups was compared with that in the model group.

### Statistical analysis

Statistical Package for the Social Sciences (version 20.0) software was applied for data analysis. The data of each group were presented as mean ± standard error (S.E.M.). One-way analysis of variance was used, and then Tukey multiple comparison test was performed. *p* < 0.05 was considered statistically significant. The graphs were drawn with GraphPad Prism version 8.0.

## Results

### Validation of HPLC method

The HPLC method was verified based on linearity, precision, stability, and reproducibility. Linearity was verified by using the standard solutions of DB. Taking the concentration as abscissa (X), and the peak area value as ordinate (Y), the linear regression equation was obtained: Y = 38.395 X—2,228.2. Data displayed that the linearity of DB in the concentration range of 0.06–0.20 mg/ml was linear (correlation coefficient r = 0.9991). Furthermore, the precision was obtained by injecting 10 µl of a standard solution six times, recording the peak area, and calculated the RSD value. Results showed that the RSD value was 0.46%, suggesting that the instrument has good precision. Besides, stability was obtained by measuring samples at 0, 3, 6, 9, 12, and 24 h, and the repeatability was determined by measuring 6 times the same sample. The results demonstrated that the RSD value of DB peak area was 2.01%, and 2.32%, separately, indicating that the test solution stability in 24 h and the method repeatability were satisfactory.

### Determination of diosbulbin B content in 95% ethanol extracts of rhizoma dioscoreae bulbiferae and processed products of angelicae sinensis radix-processed rhizoma dioscoreae bulbiferae

**FIGURE 1:**
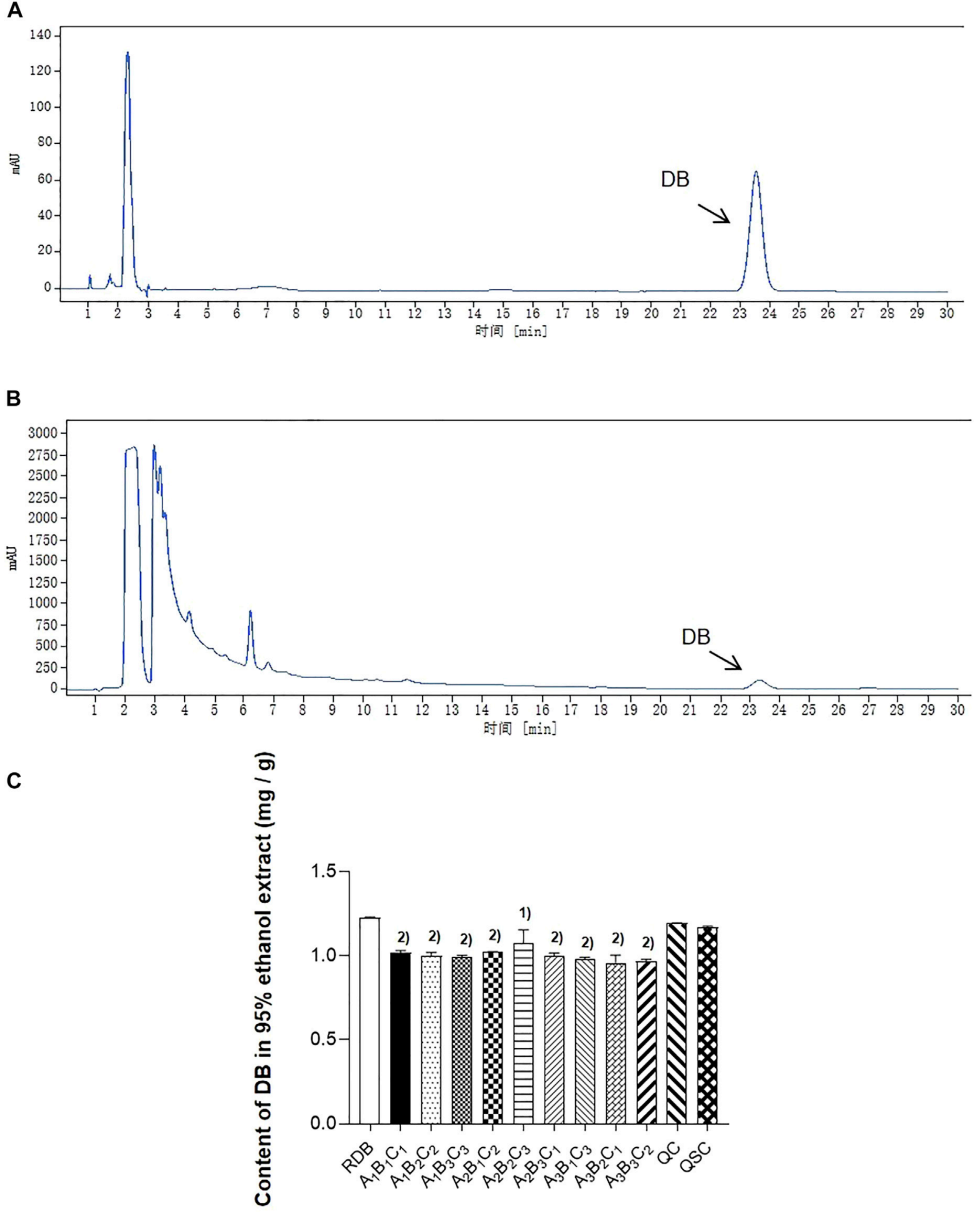
Processing with ASR alleviated the content of DB in RDB group. **(A)** The reference substance of DB in HPLC. **(B)** The sample of DB in HPLC. **(C)** The content of DB in RDB and other processed group. Data were expressed as mean ± S.E.M., with three samples in each group. 1) *p* < 0.05, 2) *p* < 0.01, compared with the RDB group.

This study firstly evaluated whether processing with ASR could effectively reduce the RDB-caused hepatotoxicity via the detection of DB content in RDB and various processed products (Figure 1; Table 2). Results showed that the DB content in the processed products (*A*_*1*_ *B*_*1*_ *C*_*1*_, *A*_*1*_ *B*_*2*_ *C*_*2*_, *A*_*1*_ *B*_*3*_ *C*_*3*_, *A*_*2*_ *B*_*1*_ *C*_*2*_, *A*_*2*_ *B*_*2*_ *C*_*3*_, *A*_*2*_ *B*_*3*_ *C*_*1*_, *A*_*3*_ *B*_*1*_ *C*_*3*_, *A*_*3*_ *B*_*2*_ *C*_*1*_, and *A*_*3*_ *B*_*3*_ *C*_*2*_) of ASR-processed RDB remarkably weakened (*p* < 0.05 or *p* < 0.01) when compared with the RDB group. Although DB content in QC and QSC products decreased somewhat, there was no discernible distinction (*p* > 0.05). In addition, based on the content of DB in RDB and processed products of ASR-processed RDB, the order from high to low was as follows: RDB product > QC product > QSC product > *A*_*2*_ *B*_*2*_ *C*_*3*_ > *A*_*2*_ *B*_*1*_ *C*_*2*_ > *A*_*1*_ *B*_*1*_ *C*_*1*_ > *A*_*1*_ *B*_*2*_ *C*_*2*_ > *A*_*2*_ *B*_*3*_ *C*_*1*_ > *A*_*1*_ *B*_*3*_ *C*_*3*_ > *A*_*3*_ *B*_*1*_ *C*_*3*_ > *A*_*3*_ *B*_*3*_ *C*_*2*_ > *A*_*3*_ *B*_*2*_ *C*_*1*_. Among them, the content of DB in the RDB product was the highest, which was 1.227 mg/g, and the *A*_*3*_ *B*_*2*_ *C*_*1*_ was the lowest, which was 0.958 mg/g.

**TABLE 2:** Determination of DB content in 95% ethanol extracts of RDB and processed products of ASR-processed RDB.

Group	DB (mg/g)	Relative rate of change (%)
RDB	1.227 ± 0.003	−
*A*_*1*_ *B*_*1*_ *C*_*1*_	1.020 ± 0.011^2)^	16.9
*A*_*1*_ *B*_*2*_ *C*_*2*_	1.001 ± 0.020^2)^	18.4
*A*_*1*_ *B*_*3*_ *C*_*3*_	0.996 ± 0.006^2)^	18.8
*A*_*2*_ *B*_*1*_ *C*_*2*_	1.021 ± 0.003^2)^	16.8
*A*_*2*_ *B*_*2*_ *C*_*3*_	1.077 ± 0.078^1)^	12.2
*A*_*2*_ *B*_*3*_ *C*_*1*_	0.998 ± 0.019^2)^	18.7
*A*_*3*_ *B*_*1*_ *C*_*3*_	0.979 ± 0.013^2)^	20.2
*A*_*3*_ *B*_*2*_ *C*_*1*_	0.958 ± 0.047^2)^	21.9
*A*_*3*_ *B*_*3*_ *C*_*2*_	0.969 ± 0.009^2)^	21.0
QC	1.191 ± 0.003	2.9
QSC	1.168 ± 0.007	4.8

### Processing with angelicae sinensis radix reversed the significantly elevated serum biochemical levels and liver cell degeneration, cytoplasmic looseness induced by rhizoma dioscoreae bulbiferae

**FIGURE 2:**
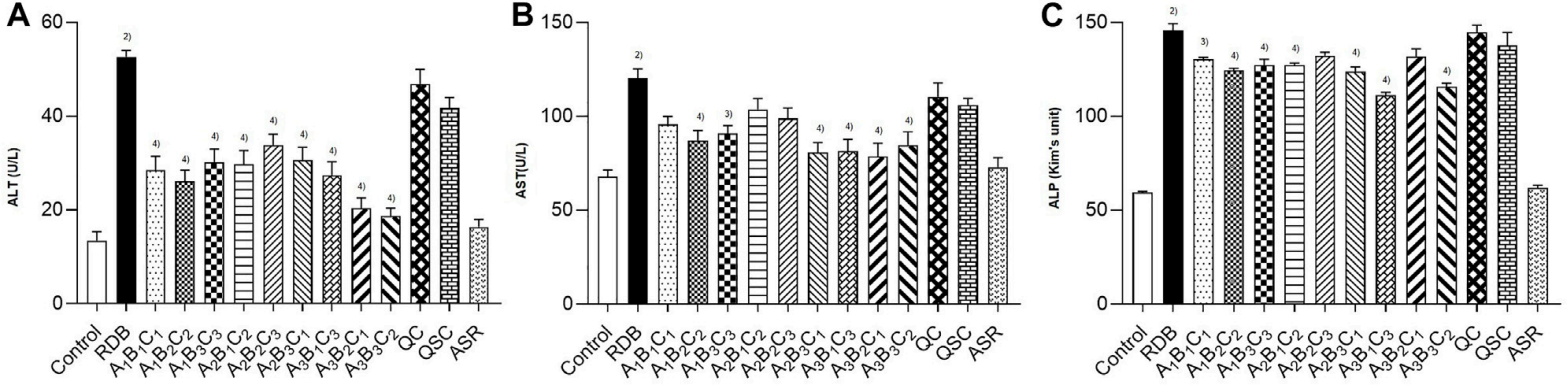
Processing with ASR inhibited the hepatotoxicity induced by RDB in mice. **(A)** Serum ALT levels. **(B)** Serum AST levels. **(C)** Serum ALP levels. Data were expressed as mean ± S.E.M., with 10 mice in each group. 1) *p* < 0.05, 2) *p* < 0.01, compared with the Control group, 3) *p* < 0.05, 4) *p* < 0.01, compared with the RDB group.

Then, this study investigated whether processing with ASR could effectively reduce the hepatotoxicity induced by RDB through the serum biochemical indicators sensitive to liver injury (Figure 2; Table 3). The results illustrated that the continuous administration of RDB remarkably elevated the levels of serum ALT, AST, and ALP by 291.8%, 77.1%, and 144.9%, respectively (all *p* < 0.01), indicating that the hepatotoxicity was induced in mice after continuous administration for 14 days. Among them, there was no obvious difference in the above-mentioned serum indexes in the ASR group (*p* > 0.05). Compared with the RDB group, the levels of serum ALT, AST, and ALP of mice decreased apparently after the intervention of five processed products, *A*_*1*_ *B*_*2*_ *C*_*2*_, *A*_*1*_ *B*_*3*_ *C*_*3*_, *A*_*2*_ *B*_*3*_ *C*_*1*_, *A*_*3*_ *B*_*1*_ *C*_*3*_, and *A*_*3*_ *B*_*3*_ *C*_*2*_ (*p* < 0.05 or *p* < 0.01). In addition, the relative change rate of serum ALT index in *A*_*3*_ *B*_*3*_ *C*_*2*_ group was the highest. In terms of the relative change rate of serum AST index, the change rate of *A*_*3*_ *B*_*2*_ *C*_*1*_ group was the highest. In terms of the relative change rate of serum ALP index, the change rate of *A*_*3*_ *B*_*1*_ *C*_*3*_ group was the highest.

**TABLE 3:** Processing with ASR reversed the significantly elevated serum biochemical levels induced by RDB.

Group	ALT (U/L^−1^)	Relative rate of change (%)	AST (U/L^−1^)	Relative rate of change (%)	ALP (Kim’s unit)	Relative rate of change (%)
Control	13.436 ± 1.890	−	67.833 ± 3.599	−	59.479 ± 0.546	−
RDB	52.638 ± 1.397^2)^	291.8	120.165 ± 5.185^2)^	77.1	145.686 ± 3.752^2)^	144.9
*A*_*1*_ *B*_*1*_ *C*_*1*_	28.500 ± 2.905^4)^	45.9	95.692 ± 4.226	20.4	130.314 ± 1.168^3)^	10.6
*A*_*1*_ *B*_*2*_ *C*_*2*_	26.069 ± 2.406^4)^	50.5	87.167 ± 5.326^4)^	27.5	124.380 ± 1.181^4)^	14.6
*A*_*1*_ *B*_*3*_ *C*_*3*_	30.115 ± 2.858^4)^	42.8	90.965 ± 4.111^3)^	24.3	127.355 ± 3.125^4)^	12.6
*A*_*2*_ *B*_*1*_ *C*_*2*_	29.712 ± 2.954^4)^	43.6	103.686 ± 5.709	13.7	127.223 ± 1.210^4)^	12.7
*A*_*2*_ *B*_*2*_ *C*_*3*_	33.775 ± 2.384^4)^	35.8	98.823 ± 5.578	17.8	132.281 ± 1.929	9.2
*A*_*2*_ *B*_*3*_ *C*_*1*_	30.558 ± 2.799^4)^	41.9	80.738 ± 5.266^4)^	32.8	123.702 ± 2.603^4)^	15.1
*A*_*3*_ *B*_*1*_ *C*_*3*_	27.338 ± 2.922^4)^	48.1	81.566 ± 6.262^4)^	32.1	111.223 ± 1.533^4)^	23.7
*A*_*3*_ *B*_*2*_ *C*_*1*_	20.391 ± 2.190^4)^	61.3	78.709 ± 6.921^4)^	34.5	131.884 ± 4.107	9.5
*A*_*3*_ *B*_*3*_ *C*_*2*_	18.658 ± 1.703^4)^	64.6	84.602 ± 7.058^4)^	29.6	115.653 ± 1.998^4)^	20.6
QC	46.885 ± 3.108	10.9	110.278 ± 7.494	8.2	144.744 ± 3.807	0.6
QSC	41.749 ± 2.222	20.7	106.019 ± 3.503	11.8	137.752 ± 6.935	5.4
ASR	16.367 ± 1.600	68.9	72.983 ± 5.127	39.3	61.942 ± 1.342	57.5

According to the analysis of the results of the above indicators, the serum transaminase levels in each processed product of ASR-processed RDB were comprehensively evaluated and analyzed by a multi-index comprehensive method (Table 4), and found that the optimized processing process of ASR-processed RDB was “processing at a mass ratio of 100:20 (RDB:ASR) and a temperature of 140°C for 10 min.” The attenuating effect of different processed products was as follows: *A*_*3*_ *B*_*2*_ *C*_*1*_ > *A*_*3*_ *B*_*3*_ *C*_*2*_ > *A*_*3*_ *B*_*1*_ *C*_*3*_ > *A*_*1*_ *B*_*2*_ *C*_*2*_ > *A*_*2*_ *B*_*3*_ *C*_*1*_ > *A*_*1*_ *B*_*3*_ *C*_*3*_ > *A*_*1*_ *B*_*1*_ *C*_*1*_ > *A*_*2*_ *B*_*1*_ *C*_*2*_ > *A*_*2*_ *B*_*2*_ *C*_*3*_.

**TABLE 4:** The results of serum T value of each group of mice of ASR-processed RDB.

Group	t _ALT_	t _AST_	t _ALP_	Comprehensive score
*A*_*1*_ *B*_*1*_ *C*_*1*_	6.161	3.600	3.521	13.282
*A*_*1*_ *B*_*2*_ *C*_*2*_	9.227	4.182	4.978	18.387
*A*_*1*_ *B*_*3*_ *C*_*3*_	7.364	5.663	3.478	16.505
*A*_*2*_ *B*_*1*_ *C*_*2*_	6.145	1.888	4.763	12.796
*A*_*2*_ *B*_*2*_ *C*_*3*_	5.331	2.192	3.691	11.214
*A*_*2*_ *B*_*3*_ *C*_*1*_	7.662	5.705	4.037	17.404
*A*_*3*_ *B*_*1*_ *C*_*3*_	7.499	4.869	9.699	22.067
*A*_*3*_ *B*_*2*_ *C*_*1*_	17.215	7.174	1.902	26.291
*A*_*3*_ *B*_*3*_ *C*_*2*_	14.286	4.165	5.792	24.243

**FIGURE 3:**
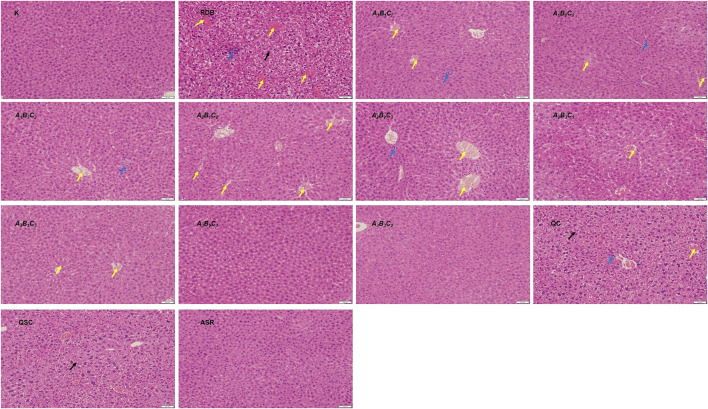
Processing with ASR improved the liver cell degeneration, cytoplasmic looseness, hepatocyte edema, inflammatory infiltration, and vascular congestion induced by RDB. HE staining (original magnifications, ×200) of liver tissues. (Black arrows indicated cytoplasmic looseness, blue arrows indicated inflammatory infiltration, and yellow arrows indicated vascular congestion).

In addition, liver histopathology (Figure 3) showed that the RDB administration resulted in a large number of hepatocytes with cytoplasmic looseness and hepatocyte edema, inflammatory infiltration, and vascular congestion in the liver of mice. However, after the intervention of processed products of ASR-processed RDB (*A*_*1*_ *B*_*2*_ *C*_*2*_, *A*_*3*_ *B*_*1*_ *C*_*3*_, *A*_*3*_ *B*_*2*_ *C*_*1*_, and *A*_*3*_ *B*_*3*_ *C*_*2*_), the hepatotoxicity was significantly improved to varying degrees. Among them, other processed products, including the QC and QSC group, only improved the toxicity induced by RDB to a certain extent, and have not yet achieved a significant effect. The results of liver pathological were consistent with or basically consistent with the serum biochemical indexes.

### Study on the detoxification mechanism of angelicae sinensis radix-Processed rhizoma dioscoreae bulbiferae under the best technology

In the early stage, we screened out the optimized processing process of ASR-processed RDB. Next, the worst detoxification technology group (*A*_*2*_ *B*_*2*_ *C*_*3*_), the intermediate processing technology (*A*_*2*_ *B*_*3*_ *C*_*1*_) group, the optimized processing (*A*_*3*_ *B*_*2*_ *C*_*1*_) group, and QC, QSC group were selected to study the molecular mechanism.

### Processing with angelicae sinensis radix reversed the significantly downregulated nrf2/heme oxygenase 1 pathway related proteins in liver caused by rhizoma dioscoreae bulbiferae

**FIGURE 4:**
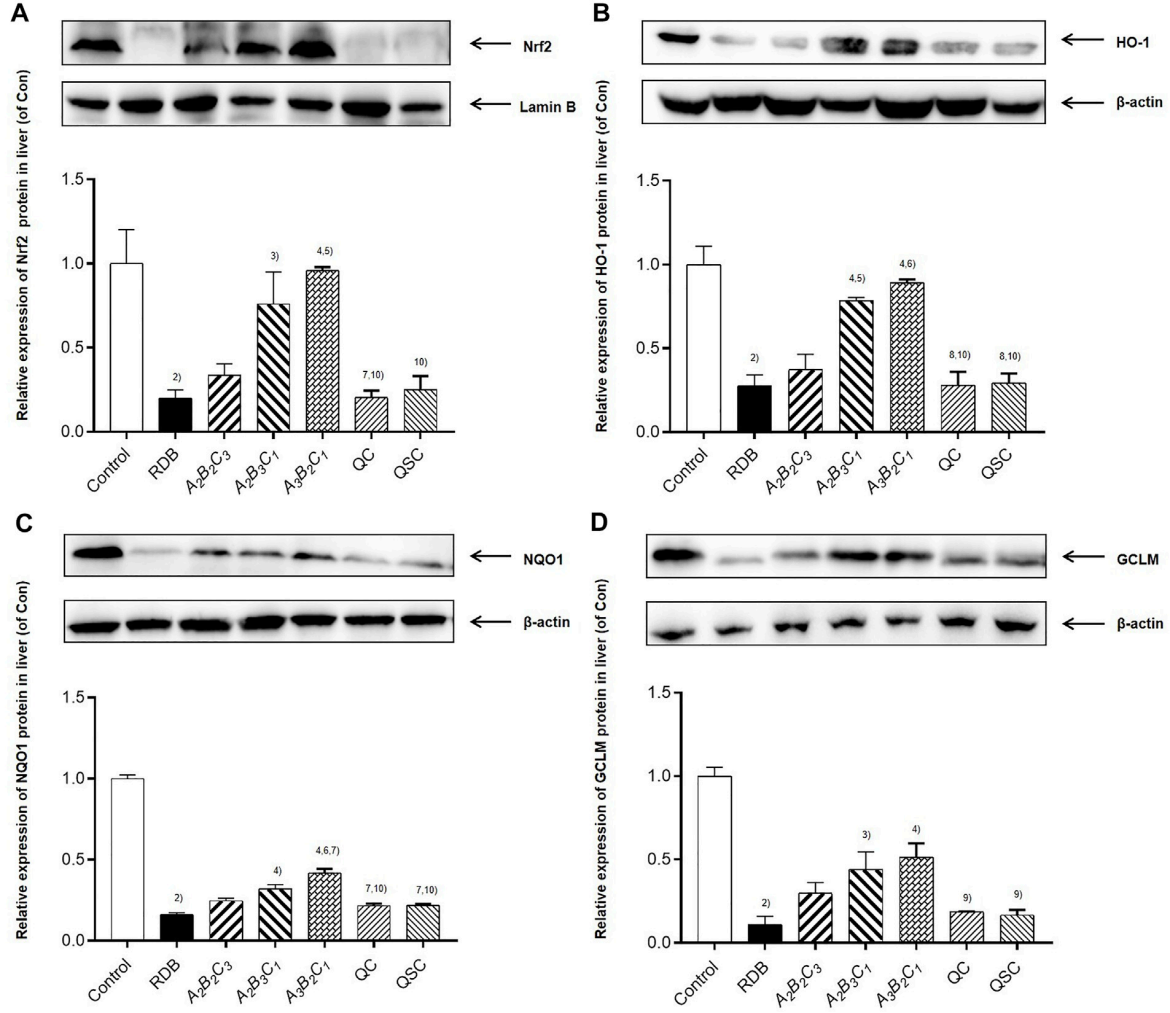
Processing with ASR reversed Nrf2-mediated antioxidant defense induced by RDB in mice. **(A)** Nrf2, **(B)** HO-1, **(C)** NQO1, **(D)** GCLM protein levels in liver determined by western blot. Data were expressed as mean ± S.E.M., with three mice in each group. 1) *p* < 0.05, 2) *p* < 0.01, compared with the Control group, 3) *p* < 0.05, 4) *p* < 0.01, compared with the RDB group, 5) *p* < 0.05, 6) *p* < 0.01, compared with the *A*_*2*_ *B*_*2*_ *C*_*3*_ group, 7) *p* < 0.05, 8) *p* < 0.01, compared with the *A*_*2*_ *B*_*3*_ *C*_*1*_ group, 9) *p* < 0.05, 10) *p* < 0.01, compared with the *A*_*3*_ *B*_*2*_ *C*_*1*_ group, 11) *p* < 0.05, 12) *p* < 0.01, compared with the QC group.

The current research explored the attenuating mechanism of ASR-processed RDB on the hepatotoxicity induced by RDB by detecting and analyzing the Nrf2/HO-1 pathway-related proteins in liver (Figure 4; Tables 5, 6). Results displayed that after continuous administration of RDB for 14 days, the protein expression levels of Nrf2/HO-1/NQO1/GCLM were remarkably down-regulated by 80.0%, 72.4%, 83.7%, and 89.1% (*p* < 0.01). After intervention with the *A*_*2*_ *B*_*3*_ *C*_*1*_ and *A*_*3*_ *B*_*2*_ *C*_*1*_ group, the Nrf2/HO-1/NQO1/GCLM expression protein levels were strikingly up-regulated by 280.0%, 184.8%, 96.9%, and 303.7%, respectively (*A*_*2*_ *B*_*3*_ *C*_*1*_ group, *p* < 0.05 or *p* < 0.01), 380.5%, 223.9%, 157.7%, and 370.6% (*A*_*3*_ *B*_*2*_ *C*_*1*_ group, *p* < 0.01). After the intervention of QC, QSC, and *A*_*2*_ *B*_*2*_ *C*_*3*_ groups, although the above indicators showed an upward trend, there was no obvious distinction (*p* > 0.05). In addition, in terms of the relative change rate of Nrf2/HO-1/NQO1/GCLM, *A*_*3*_ *B*_*2*_ *C*_*1*_ group had the highest rate of change.

**TABLE 5:** Processing with ASR reversed the significantly down-regulated Nrf2/HO-1 pathway related proteins in liver caused by RDB.

Group	Nrf2/Lamin B	Relative rate of change (%)	HO-1/β-actin	Relative rate of change (%)
Control	1.000 ± 0.202	−	1.000 ± 0.109	−
RDB	0.200 ± 0.049^2)^	80.0	0.276 ± 0.066^2)^	72.4
*A*_*2*_ *B*_*2*_ *C*_*3*_	0.337 ± 0.067	68.5	0.373 ± 0.091	35.1
*A*_*2*_ *B*_*3*_ *C*_*1*_	0.760 ± 0.190^3)^	280.0	0.786 ± 0.018^4,5)^	184.8
*A*_*3*_ *B*_*2*_ *C*_*1*_	0.961 ± 0.018^4,5)^	380.5	0.894 ± 0.018^4,6)^	223.9
QC	0.203 ± 0.042^7,10)^	1.5	0.280 ± 0.079^8,10)^	1.4
QSC	0.252 ± 0.081^10)^	26.0	0.292 ± 0.057^8,10)^	5.8

**TABLE 6:** Processing with ASR reversed the significantly down-regulated NQO1/GCLM pathway related proteins in liver caused by RDB.

Group	NQO1/β-actin	Relative rate of change (%)	GCLM/β-actin	Relative rate of change (%)
Control	1.000 ± 0.024	−	1.000 ± 0.054	−
RDB	0.163 ± 0.010^2)^	83.7	0.109 ± 0.050^2)^	89.1
*A*_*2*_ *B*_*2*_ *C*_*3*_	0.248 ± 0.015	52.1	0.298 ± 0.064	173.4
*A*_*2*_ *B*_*3*_ *C*_*1*_	0.321 ± 0.026^4)^	96.9	0.440 ± 0.106^3)^	303.7
*A*_*3*_ *B*_*2*_ *C*_*1*_	0.420 ± 0.024^4,6,7)^	157.7	0.513 ± 0.084^4)^	370.6
QC	0.217 ± 0.013^7,10)^	33.1	0.186 ± 0.003^9)^	70.6
QSC	0.221 ± 0.007^7,10)^	35.6	0.167 ± 0.032^9)^	53.2

### Processing with angelicae sinensis radix remarkably reversed hepatic lipid peroxidation and enhanced antioxidant levels in the liver of mice caused by rhizoma dioscoreae bulbiferae

**FIGURE 5:**
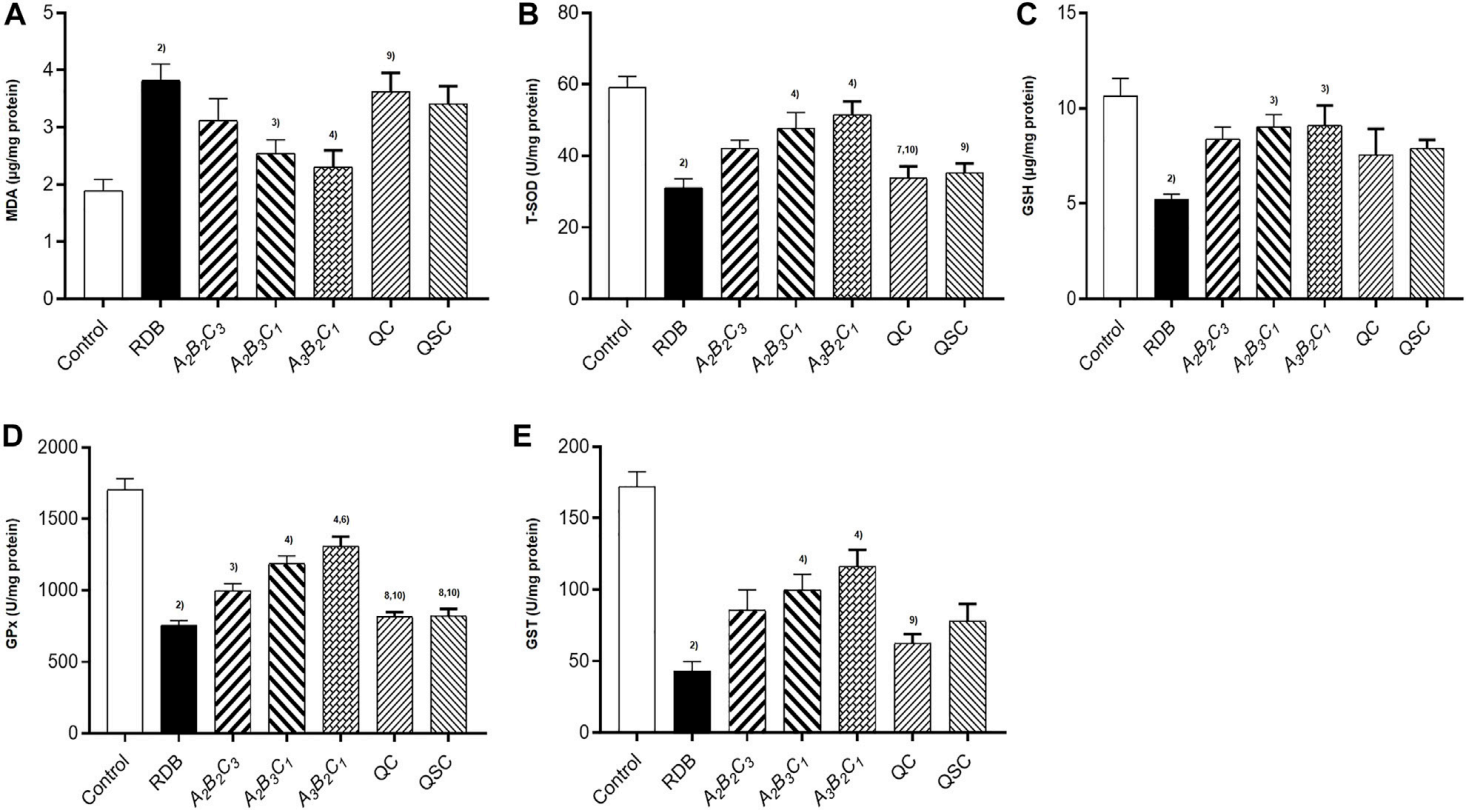
Processing with ASR ameliorated hepatic oxidative stress induced by RDB in mice. **(A)** Hepatic MDA levels. **(B)** Hepatic T-SOD activity. **(C)** Hepatic GSH levels. **(D)** Hepatic GPx activity. **(E)** Hepatic GST activity. Data were expressed as mean ± S.E.M., with 10 mice in each group. 1) *p* < 0.05, 2) *p* < 0.01, compared with the Control group, 3) *p* < 0.05, 4) *p* < 0.01, compared with the RDB group, 5) *p* < 0.05, 6) *p* < 0.01, compared with the *A*_*2*_ *B*_*2*_ *C*_*3*_ group, 7) *p* < 0.05, 8) *p* < 0.01, compared with the *A*_*2*_ *B*_*3*_ *C*_*1*_ group, 9) *p* < 0.05, 10) *p* < 0.01, compared with the *A*_*3*_ *B*_*2*_ *C*_*1*_ group, 11) *p* < 0.05, 12) *p* < 0.01, compared with the QC group.

Next, the current research further evaluated the detoxification mechanism of ASR-processed RDB through the detection and analysis of lipid peroxidation and antioxidant levels in liver (Figure 5; Tables 7, 8). Data displayed that the MDA level was effectively augmented by 102.1% (*p* < 0.01) in liver, and the T-SOD, GSH, GPx, and GST levels were strikingly decreased by 47.7%, 51.1%, 55.6%, and 75.0% (*p* < 0.01), after 14 days of continuous administration of RDB. After the intervention of *A*_*2*_ *B*_*3*_ *C*_*1*_ group and *A*_*3*_ *B*_*2*_ *C*_*1*_ group, the MDA level was strikingly alleviated by 33.3% and 39.6% (*p* < 0.05 or *p* < 0.01), and the levels of T-SOD, GSH, GPx, and GST were significantly augmented by 54.0%, 73.4%, 56.6%, and 132.2% (*A*_*2*_ *B*_*3*_ *C*_*1*_ group, *p* < 0.05 or *p* < 0.01), 66.0%, 74.9%, 73.1%, and 170.2% (*A*_*3*_ *B*_*2*_ *C*_*1*_ group, *p* < 0.05 or *p* < 0.01), separately. However, the administration of *A*_*2*_ *B*_*2*_ *C*_*3*_ group only remarkably increased the level of GPx in liver (*p* < 0.05). In addition, after the treatment with QC, QSC, and *A*_*2*_ *B*_*2*_ *C*_*3*_ group, although the MDA level showed a decreasing trend, the three indicators of T-SOD, GSH, and GST showed an increasing trend, there was no obvious difference (*p* < 0.05). Besides, for the relative change rates of MDA, T-SOD, GSH, GPx, and GST indicators mentioned above, *A*_*3*_ *B*_*2*_ *C*_*1*_ group was the highest.

**TABLE 7:** Processing with ASR remarkably reversed hepatic lipid peroxidation and enhanced antioxidant levels in the liver of mice caused by RDB.

Group	MDA (μg/mg protein)	Relative rate of change (%)	T-SOD (U/mg protein)	Relative rate of change (%)
Control	1.890 ± 0.200	−	59.260 ± 2.977	−
RDB	3.819 ± 0.286^2)^	102.1	31.012 ± 2.535^2)^	47.7
*A*_*2*_ *B*_*2*_ *C*_*3*_	3.117 ± 0.387	18.4	42.094 ± 2.341	35.7
*A*_*2*_ *B*_*3*_ *C*_*1*_	2.547 ± 0.236^3)^	33.3	47.772 ± 4.402^4)^	54.0
*A*_*3*_ *B*_*2*_ *C*_*1*_	2.307 ± 0.290^4)^	39.6	51.488 ± 3.736^4)^	66.0
QC	3.626 ± 0.326^9)^	5.1	33.849 ± 3.237^7,10)^	9.1
QSC	3.413 ± 0.305	10.6	35.329 ± 2.604^9)^	13.9

**TABLE 8:** Processing with ASR remarkably enhanced levels of glutathione antioxidant enzymess in the liver of mice caused by RDB.

Group	GSH (μg/mg protein)	Relative rate of change (%)	GPx (U/mg protein)	Relative rate of change (%)	GST (U/mg protein)	Relative rate of change (%)
Control	10.642 ± 0.923	−	1704.978 ± 78.231	−	172.167 ± 10.390	−
RDB	5.205 ± 0.292^2)^	51.1	756.807 ± 32.897^2)^	55.6	43.012 ± 6.793^2)^	75.0
*A*_*2*_ *B*_*2*_ *C*_*3*_	8.393 ± 0.632	61.2	999.935 ± 45.373^3)^	32.1	85.613 ± 14.386	99.0
*A*_*2*_ *B*_*3*_ *C*_*1*_	9.025 ± 0.647^3)^	73.4	1,185.429 ± 55.245^4)^	56.6	99.866 ± 10.838^4)^	132.2
*A*_*3*_ *B*_*2*_ *C*_*1*_	9.101 ± 1.047^3)^	74.9	1,310.261 ± 66.209^4,6)^	73.1	116.210 ± 11.710^4)^	170.2
QC	7.556 ± 1.358	45.2	815.303 ± 32.070^8,10)^	7.7	62.427 ± 6.457^9)^	45.1
QSC	7.910 ± 0.429	52.0	820.837 ± 49.627^8,10)^	8.5	77.906 ± 11.938	81.1

### Processing with angelicae sinensis radix conserved the antitussive and expectorant efficacy of rhizoma dioscoreae bulbiferae

**FIGURE 6:**
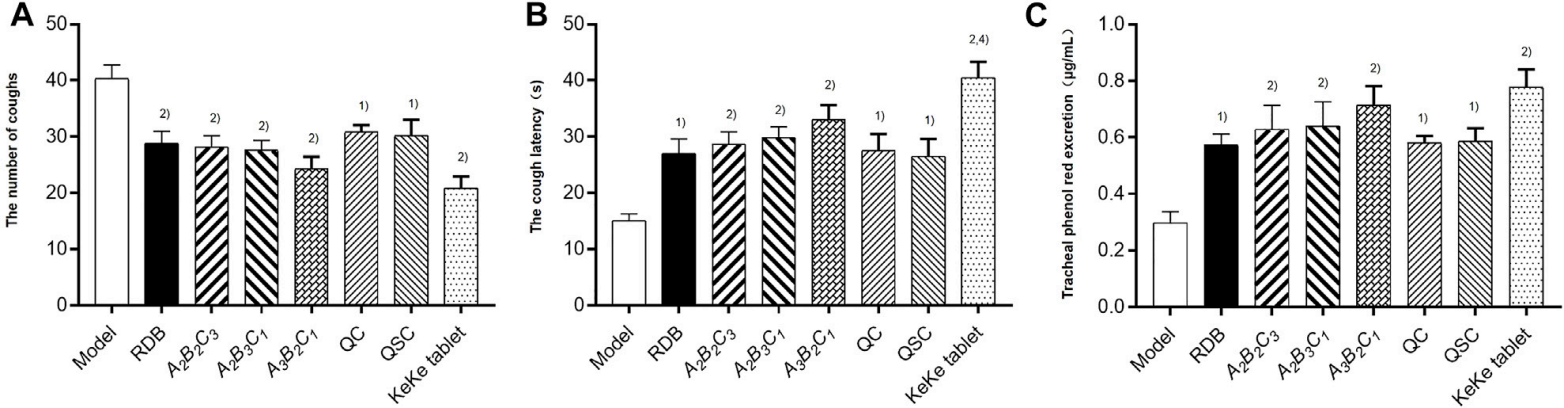
Processing with ASR conserved the antitussive and expectorant efficacy of RDB in mice. **(A)** The number of coughs. **(B)** The cough latency. **(C)** Tracheal phenol red excretion. Data were expressed as mean ± S.E.M., with 10 mice in each group. 1) *p* < 0.05, 2) *p* < 0.01, compared with the Model group, 3) *p* < 0.05, 4) *p* < 0.01, compared with the RDB group, 5) *p* < 0.05, 6) *p* < 0.01, compared with the *A*_*3*_ *B*_*2*_ *C*_*1*_ group.

Finally, this study evaluated the efficacy of processing with ASR on the antitussive and expectorant effects of RDB through ammonia-induced cough model and the excretion of phenol red in the trachea (Figure 6; Table 9). Experimental data displayed that compared with the model group, after continuous administration of RDB and various processed products (*A*_*2*_ *B*_*2*_ *C*_*3*_, *A*_*2*_ *B*_*3*_ *C*_*1*_, *A*_*3*_ *B*_*2*_ *C*_*1*_, QC, and QSC) of ASR-processed RDB, and the positive drug Keke Tablets, the cough latency and the amount of phenol red excretion in the trachea of mice were remarkably prolonged, and the number of coughs was effectively reduced (*p* < 0.05 or *p* < 0.01), and the cough relieving rates of processed products and Keke tablets were 28.5%, 30.0%, 31.5%, 39.7%, 23.6%, 25.1%, and 48.1%, respectively. The relative percentages of phenol red concentrations were 92.0%, 110.4%, 114.4%, 139.5%, 94.3%, 96.0%, and 160.2%, respectively. Compared with the RDB group, the cough latency of mice in the positive drug Keke tablet group was significantly prolonged (*p* < 0.01). In addition, there was no obvious distinction in cough latency, the number of coughs, or phenol red excretion between *A*_*3*_ *B*_*2*_ *C*_*1*_ and Keke tablet group (*p* > 0.05).

**TABLE 9:** Processing with ASR conserved the antitussive and expectorant efficacy of RDB.

Group	The number of coughs	The cough latency (s)	The cough relieving rates (%)	Phenol red concentration (μg/ml^−1)^	Relative rate of change (%)
Model	40.300 ± 2.468	15.060 ± 1.192	−	0.299 ± 0.039	−
RDB	28.800 ± 2.185^2)^	27.004 ± 2.610^1)^	28.5	0.574 ± 0.038^1)^	92.0
*A*_*2*_ *B*_*2*_ *C*_*3*_	28.200 ± 2.004^2)^	28.736 ± 2.110^2)^	30.0	0.629 ± 0.085^2)^	110.4
*A*_*2*_ *B*_*3*_ *C*_*1*_	27.600 ± 1.752^2)^	29.887 ± 1.887^2)^	31.5	0.641 ± 0.086^2)^	114.4
*A*_*3*_ *B*_*2*_ *C*_*1*_	24.300 ± 2.150^2)^	33.109 ± 2.522^2)^	39.7	0.716 ± 0.067^2)^	139.5
QC	30.800 ± 1.245^1)^	27.529 ± 2.936^1)^	23.6	0.581 ± 0.025^1)^	94.3
QSC	30.200 ± 2.832^1)^	26.492 ± 3.104^1)^	25.1	0.586 ± 0.047^1)^	96.0
Keke Tablets	20.900 ± 2.025^2)^	40.515 ± 2.850^2,4)^	48.1	0.778 ± 0.063^2)^	160.2

## Discussion

The detoxification technology of processing with ASR on RDB-induced hepatotoxicity was evaluated in the current research, and confirmed that the optimized processing process of ASR-processed RDB is “processing at a mass ratio of 100:20 (RDB:ASR) and a temperature of 140°C for 10 min,” and the processing detoxification mechanism involved enhancing the level of Nrf2-mediated antioxidant defense.

As the main toxic component of RDB, if the DB content increased significantly, it often indicates toxicity is induced (Wang et al., 2012). Therefore, the DB content in processed products of ASR-processed RDB was determined to preliminarily optimize the processing and detoxification technology*.* Results displayed that DB content in RDB and processed products of ASR-processed RDB all decreased to a certain extent, and the DB content in the processed products of *A*_*3*_ *B*_*2*_ *C*_*1*_ decreased the most, which was 0.958 mg/g, with the reversal rate of 21.9%, suggested that processing with ASR reduced the toxicity induced by RDB, and the *A*_*3*_ *B*_*2*_ *C*_*1*_ group had the best attenuating effect.

Under physiological conditions, there were only trace amounts of ALT and AST in the blood (Yuan et al., 2018; Guo et al., 2021). When hepatocytes were damaged, large amounts of ALT and AST will enter the blood, resulting in an obvious augment in serum ALT and AST levels (Salahshoor et al., 2018; Zhang et al., 2018; Wang L. et al., 2019; Kaya et al., 2021; Li H. et al., 2021). Therefore, serum ALT and AST indicators can be used to reflect the severity of liver damage (Gou et al., 2021; Jafaripour et al., 2021). In view of this, the research investigated the attenuating effect of processing with ASR on RDB-caused liver damage *via* measuring the serum ALT, AST, and ALP levels. Results found that RDB caused a noticeable increase in the levels of serum ALT, AST, and ALP by 291.8%, 77.1%, and 144.9%, respectively, indicating that the administration of RDB indeed induced liver toxicity, which was consistent or substantially consistent with some studies (Wang et al., 2010; Ma et al., 2014; Sheng et al., 2014). It was worth noting that the elevated serum ALT, AST, and ALP levels were remarkably reversed after administration of the five processed products (*A*_*1*_ *B*_*2*_ *C*_*2*_, *A*_*1*_ *B*_*3*_ *C*_*3*_, *A*_*2*_ *B*_*3*_ *C*_*1*_, *A*_*3*_ *B*_*1*_ *C*_*3*_, and *A*_*3*_ *B*_*3*_ *C*_*2*_, respectively). The above results indicated that processing with ASR could inhibit the liver injury induced by RDB*.* However, for a single index of ALT, the optimized processing technology of ASR-processed RDB was *A*_*3*_ *B*_*3*_ *C*_*2*_ group. For AST, *A*_*3*_ *B*_*2*_ *C*_*1*_ group was the optimized detoxification technology. For ALP, *A*_*3*_ *B*_*1*_ *C*_*3*_ group was the optimized detoxification technology. Subsequently, with the help of multi-index comprehensive evaluation and analysis method, the above three indicators were comprehensively evaluated and analyzed, and the optimized processing technology of ASR-processed RDB was selected as the eighth group, namely, “processing at a mass ratio of 100:20 (RDB:ASR) and a temperature of 140°C for 10 min.” After that, in order to better judge the therapeutic effect of processing with ASR on liver injury induced by RDB, the pathological changes of liver tissues were observed by HE staining. Results showed that the processed products of ASR-processed RDB could significantly reduce cytoplasmic looseness and hepatocyte edema, inflammatory infiltration, and vascular congestion.

TCM believes that the main reason for the toxicity of RDB may be that it is bitter and cold, the strong attack and dispersion of RDB, the long-term medication for cancer treatment, the vulnerability and consumption of righteousness, and the syndrome of liver and kidney yin deficiency, phlegm and blood stasis (Fan et al., 2014). So why processing with ASR could effectively reduce the hepatotoxicity induced by RDB. It is preliminarily speculated that this may be related to its inherent composition, nature, flavor, and meridian tropism. ASR is sweet in taste, warm in nature, and returns to the meridians of liver, heart and spleen, which can not only support righteousness but also nourish Yin and blood. The material basis for reducing the toxicity of ASR-processed RDB may be related to its inherent “bitter and sweet phase” (Li et al., 2013).

Numerous studies have shown that oxidative stress occupied prominent values in the pathogenesis of hepatotoxicity (Guo et al., 2019; Lv et al., 2020; Temel et al., 2020; Jung et al., 2021; Lim et al., 2021). When the body’s oxidation and anti-oxidation are out of balance, excess reactive oxygen species will be produced, resulting in a state of oxidative stress, lipid peroxidation of cell membranes, and abnormal organelle function (Bao et al., 2010; Glade and Meguid, 2017; Hussein et al., 2020). As a core transcription factor of anti-oxidation, Nrf2 activation could mediate the expression of downstream antioxidant indicators (Liao et al., 2019; Chen et al., 2020). In addition, existing studies displayed that Nrf2 knockout mice are more likely to aggravate liver damage and induce oxidative stress response in liver than wild-type mice (Shi et al., 2018; Wei et al., 2018; Jin et al., 2019; Lyu et al., 2020), and the use of Nrf2 activator could alleviate the disease caused by oxidative stress (Kim et al., 2017; Kim et al., 2020). Moreover, most studies indicated that the expression level of Nrf2 in the liver was reflected by detecting the expression of Nrf2 in the nucleus (Lv et al., 2019; Wu et al., 2019; Wang et al., 2021; Zhou et al., 2021). As the final product of lipid peroxidation, the content of MDA could reflect the damage degree of lipid peroxidation (Jiang W. et al., 2021). T-SOD, GSH, and GPx are liver oxidation active enzymes, which could remove peroxides and free radicals, alleviate liver damage and protect hepatocytes (Li et al., 2018; Xie et al., 2020b; Li Z. et al., 2021; Yang et al., 2021), and were considered as important indicators reflecting hepatic oxidative damage (Park et al., 2019; Chen T. et al., 2021). Consequently, after confirming that processing with ASR could significantly improve the liver toxicity induced by RDB, the potential molecular mechanism of ASR-processed RDB to reduce its liver toxicity was evaluated by detecting and analyzing Nrf2 and its downstream-related molecules.

Similar to the results of previous studies (Wang et al., 2010; Ma et al., 2013), this study showed that the administration of RDB significantly downregulated the expression level of Nrf2 protein in liver, and decreased the expression of HO-1, NQO1, GCLM, T-SOD, GSH, and GPx, GST antioxidant signal molecules downstream of Nrf2, suggesting that RDB-induced hepatotoxicity involves weak antioxidant defense. Notably, data showed that the processed products of ASR-processed RDB effectively improved the abnormality of Nrf2 and its downstream molecules, manifesting that the detoxification mechanism of the processed products of ASR-processed RDB involved enhancing the Nrf2 signaling pathway. Other results also showed that the administration of RDB caused liver lipid peroxidation damage in mice, which was mainly manifested in the significant increase in MDA level, and similar to the study (Niu et al., 2014). Satisfyingly, the intervention of different processed products significantly reduced the MDA level, indicating that the inhibition of liver lipid peroxidation damage was also involved in its detoxification mechanism. However, it was worth noting that this study did not use target diagnostic agents (such as inhibitors and agonists) and gene knockout technology to explore the in-depth mechanism of processing with ASR to reduce liver toxicity induced by RDB, which was the limitation of the study and an important research direction in the future.

The above results showed that processing with ASR could effectively improve RDB*-*caused liver damage, so is it possible to reduce the hepatotoxicity, simultaneously, its curative effect is not reduced or even enhanced? Taking into account that RDB has good antitussive and expectorant efficacy, then, the present research mainly explored the antitussive and expectorant efficacy of RDB through the ammonia-induced cough model and the tracheal phenol red excretion experiment. The results showed that RDB and the products of ASR-processed RDB (processed products: *A*_*2*_ *B*_*2*_ *C*_*3*_, *A*_*2*_ *B*_*3*_ *C*_*1*_, and *A*_*3*_ *B*_*2*_ *C*_*1*_), and the products of QC, QSC apparently augmented the cough latency and phenol red excretion of trachea in cough mice induced by ammonia to varying degrees, and reduced the number of coughs caused by ammonia, which suggested that the above-mentioned processed products have good efficacy in relieving cough and eliminating phlegm. However, compared with the two processed products of QC and QSC mentioned above, they did not remarkably improve the RDB-induced hepatotoxicity, which suggested that the two processed products of QC and QSC only have the preservation effect and did not have the effect of reducing toxicity. As for the processed products (*A*_*2*_ *B*_*2*_ *C*_*3*_, *A*_*2*_ *B*_*3*_ *C*_*1*_, and *A*_*3*_ *B*_*2*_ *C*_*1*_), they not only conserved the antitussive and expectorant effect of RDB, but also improved the hepatotoxicity induced by RDB to a certain extent. In addition, from the results of serum transaminase level and the degree of pathological injury of liver tissue in mice, the *A*_*3*_ *B*_*2*_ *C*_*1*_ group significantly reduced the levels of serum ALT, AST, and ALP and pathological injury of liver in mice, and the reversal rate of each index in *A*_*3*_ *B*_*2*_ *C*_*1*_ group was the highest compared with other groups, which indicated that this group had the best detoxification effect, that was, the optimized processing technology group. From the results of antitussive and expectorant efficacy, the *A*_*3*_ *B*_*2*_ *C*_*1*_ group significantly increased the cough latency and tracheal phenol red excretion, and reduced the number of coughs, and the reversal rate of each index in *A*_*3*_ *B*_*2*_ *C*_*1*_ group was the highest compared with other groups, which indicated that this group had the best treatment effect, that was, the optimized processing technology group. To sum up, the optimized processing process of ASR-processed RDB was “processing at a mass ratio of 100:20 (RDB:ASR) and a temperature of 140°C for 10 min”.

In conclusion, the current study confirmed that the optimized processing process of ASR-processed RDB was “processing at a mass ratio of 100:20 (RDB:ASR) and a temperature of 140°C for 10 min,” and the processing detoxification mechanism involved enhancing the level of Nrf2-mediated antioxidant defense in liver. This study provided a certain experimental support for the promotion and application, and laid a preliminary foundation for the establishment of the detoxification system of RDB.

## Data Availability

The original contributions presented in the study are included in the article/supplementary material, further inquiries can be directed to the first author or the corresponding author.

## Abbreviations

ALP, Alkaline phosphatase; ALT, Alanine aminotransferase; ASR, Angelicae Sinensis Radix; AST, Aspartate aminotransferase; GCLM, Glutamatecysteine ligase; GPx, Glutathione peroxidase; GSH, Reduced glutathione; GST, Glutathione S-transferase; HO-1, Heme Oxygenase-1; HPLC, High performance liquid chromatography; MDA, Malondialdchydc; NQO1, Quinone oxidoreductase 1; Nrf2, Nuclear Factor-E2-related factor 2; RDB, Rhizoma Dioscoreae Bulbiferae; T-SOD, Total superoxide dismutase.
